# Potential Functions of Gem-Associated Protein 2-Like Isoform X1 in the Oriental River Prawn *Macrobrachium nipponense*: Cloning, qPCR, In Situ Hybridization, and RNAi Analysis

**DOI:** 10.3390/ijms20163995

**Published:** 2019-08-16

**Authors:** Shubo Jin, Yuning Hu, Hongtuo Fu, Sufei Jiang, Yiwei Xiong, Hui Qiao, Wenyi Zhang, Yongsheng Gong, Yan Wu

**Affiliations:** 1 Key Laboratory of Freshwater Fisheries and Germplasm Resources Utilization, Ministry of Agriculture, Freshwater Fisheries Research Center, Chinese Academy of Fishery Sciences, Wuxi 214081, China; 2 Wuxi Fisheries College, Nanjing Agricultural University, Wuxi 214081, China

**Keywords:** *Macrobrachium nipponense*, Gem-associated protein 2-like isoform X1, male sexual differentiation and development, in situ hybridization, RNAi

## Abstract

Gem-associated protein 2-like isoform X1 (*GEM*) was previously predicted to be involved in the sexual development of male *Macrobrachium nipponense*. In this study, we analyze the *GEM* functions in depth using quantitative polymerase chain reaction (qPCR), in situ hybridization, and RNA interference (RNAi). The full-length *Mn-GEM* cDNA sequence was 1018 base pairs (bp) long with an open reading frame of 777 bp encoding 258 amino acids. qPCR analysis of *Mn-GEM* in different tissues and developmental stages showed that *Mn-GEM* was highly expressed in the gonad and from post-larval developmental stage day 5 (PL5) to PL15, which indicated that *GEM* has potential roles in gonad differentiation and development in *M*. *nipponense*. In situ hybridization and qPCR analysis of various stages of the reproductive cycle of the testis and ovary indicated that *GEM* may promote spermatid development and gametogenesis in *M*. *nipponense*. After injecting with double-stranded RNA (dsRNA) of *Mn-GEM*, mRNA expression of Mn-insulin-like androgenic gland hormone (*Mn-IAG*) and the content of testosterone increased with the decrease of *Mn-GEM* expression, indicating that *GEM* has negative effects on the male sexual differentiation and development in *M*. *nipponense*. Results of this study highlight the functions of *GEM* in *M*. *nipponense*, which can be applied to future studies of male sexual development in *M*. *nipponense* and other crustacean species.

## 1. Introduction

The oriental river prawn *Macrobrachium nipponense* (Crustacea; Decapoda; Palaemonidae) is widely distributed in Asian countries [1,2,3,4,5]. The annual aquaculture production in 2016 was 205,010 tons in China [6]. Male *M*. *nipponense* are preferred over females in *M*. *nipponense* aquaculture, because they grow faster and reach larger size at harvest. Production of all male progeny on a commercial scale is the long-term goal in *M*. *nipponense* aquaculture. Therefore, a full understanding of the mechanism of sex differentiation of *M*. *nipponense* is urgently needed.

The androgenic gland in crustaceans produces hormones and drives male sexual differentiation in crustacean species. It plays essential roles in the establishment of male sexual characteristics and the development of the testes [7]. In studies of *Macrobrachium rosenbergii*, neo-females were generated by ablating the androgenic gland of male prawns, and mating of neo-females with normal males generated all male progeny [7,8,9]. Thus, the genes in the androgenic gland may be the key to understanding the mechanism of male differentiation and development of *M*. *nipponense*. The androgenic gland transcriptome and miRNA library for *M*. *nipponense* were reported previously [10,11], and genes from the androgenic gland transcriptome have been shown be involved in sex differentiation in *M*. *nipponense* [12,13,14,15].

Quantitative proteomic profiling analysis of the androgenic gland from both non-reproductive and reproductive seasons was performed in *M. nipponense* using the isobaric tags for relative and absolute quantitation (iTRAQ) technique [16]. Gem-associated protein 2-like isoform X1 (*GEM*) was differentially expressed between the two seasons. The expressions of *GEM* were further verified in the testes, ovaries, and androgenic gland using quantitative polymerase chain reaction (qPCR), which showed highest expression in the androgenic gland [16]. The function of *GEM* is still not well identified in other species, but *GEM* is predicted to play essential roles in male sex differentiation and development in *M. nipponense*. The potentially novel functions of *GEM* in male sexual differentiation and development need to be identified to better understand the mechanism of sex determination and differentiation in *M*. *nipponense*.

The goal of this study is to analyze the functions of *GEM* in *M*. *nipponense* and to identify their potentially important roles in sex differentiation and development. qPCR was used to evaluate the mRNA expression patterns of *Mn-GEM* in different tissues and developmental stages and in the reproductive cycle of ovaries and testes. The functions of *GEM* were further determined using in situ hybridization and RNA interference (RNAi). Results of this study will be applicable to future studies of male sexual development in *M*. *nipponense* and other crustacean species.

## 2. Results

### 2.1. Sequence Analysis

**Figure 1:**
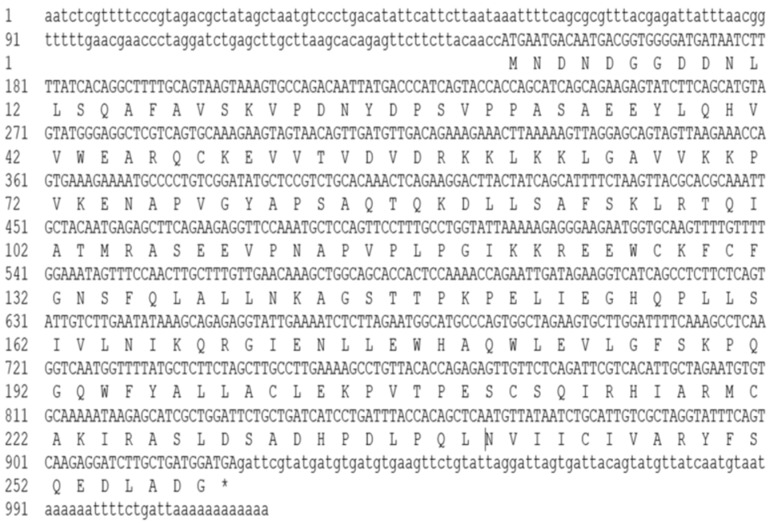
Nucleotide and deduced amino acid sequence of *Mn-GEM*. The nucleotide sequence is displayed in the 5′–3′ directions and numbered at the left. The deduced amino acid sequence is shown in a single capital letter amino acid code. 3′ UTR (untranslated region) and 5′ UTR are shown with lowercase letters. Codons are numbered at the left with the methionine (ATG) initiation codon, an asterisk (*) denotes the termination codon (TGA).

The full-length *Mn-GEM* cDNA sequence was 1018 base pairs (bp) long and was submitted to GenBank with accession no. MH817847. The open reading frame was 777 bp long, encoding for 258 amino acids. The 5′ untranslated region (UTR) was 147 bp in length and the 3′ UTR was 94 bp long (Figure 1). The molecular weight and theoretical isoelectric point of the protein were 28.68 kDa and 6.53, respectively. One SIP1 superfamily was detected in the protein sequence of *Mn-GEM*. Five O-GlcNAc sites were predicted in *Mn-GEM* by the YinOYang program. Six membrane helix and two membrane strand were detected, respectively.

**Figure 2:**
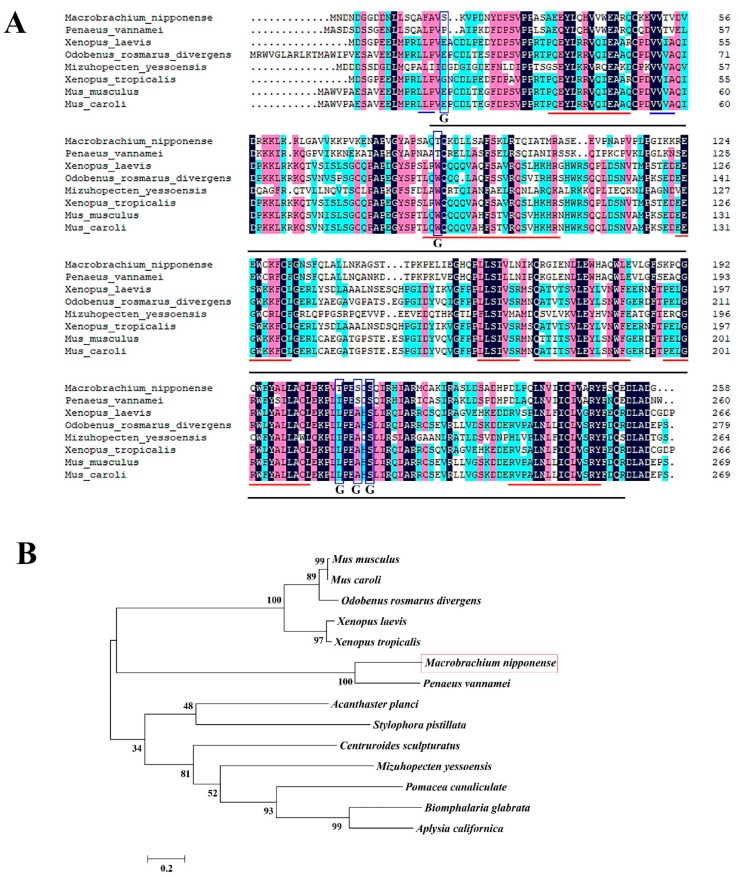
**(A)** The alignment of amino acid sequences of Gem-associated protein 2-like isoform X1 (*GEM*). Black line indicated SIP1 superfamily; Red lines indicated membrane helix; Blue lines indicated membrane strand; Black rectangles indicated the five potential O-GlcNAc sites. (**B**) The phylogenetic tree of *GEM* from different organisms based on amino acid sequence comparisons. Species names and types of *GEM* are listed on the right of the tree. Red rectangles indicated *M. nipponense*.

Table 1 lists the species used for the *GEM* amino acid sequence BLAST analysis. According to the BLASTP similarity comparisons, *Mn-GEM* has the highest sequence similarity (70%) with *GEM* of *Penaeus vannamei*. Similarities between *Mn-GEM* and *GEM* in other species was 42–47%, while the query coverage reached 98% (Figure 2A). To analyze the evolutionary relationship between *Mn-GEM* and other well-defined *GEM* sequences in NCBI, we used MEGA X followed by the maximum-likelihood method to construct a condensed phylogenetic tree based on the completed amino acid sequences of *GEM*. The phylogenetic tree showed that the amino acid sequence from *M*. *nipponense* had the closest evolutionary relationship with that of *P. vannamei* and relatively close evolutionary relationships with that of species of *Mus* and *Xenopus* (Figure 2B).

### 2.2. Expression Analysis in Different Tissues and Developmental Stages

**Figure 3:**
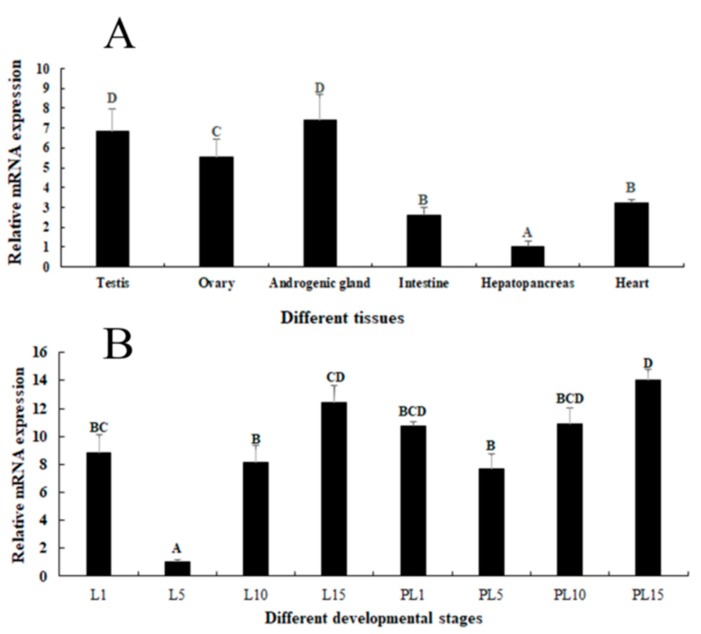
Expression characterization of *Mn-GEM* in different tissues and developmental stages. The amount of *Mn-GEM* mRNA was normalized to the eukaryotic translation initiation factor 5A (EIF) transcript level. Data are shown as mean +SD (standard deviation) of tissues from three separate individuals. Capital letters indicate expression difference between different samples. (**A**) Expression characterization in different tissues. (**B**) Expression characterization in different developmental stages.

Tissue distribution may reflect the physiological function of a protein. qPCR was used to evaluate the tissue distribution of *Mn-GEM* mRNA (Figure 3A). *Mn-GEM* expression was highest in the androgenic gland, followed by the testes and ovaries, and expression did not differ significantly between testes and ovaries (*p* > 0.05). Expression levels in the intestine and heart were low, and expression was lowest in the hepatopancreas. The *Mn-GEM* expressions in androgenic gland, testis, and ovary were 7.41-fold, 6.85-fold, and 5.56-fold higher than that in the hepatopancreas, respectively.

*Mn-GEM* expression reached the bottom at larval developmental stage day 5 (L5) and showed significant difference with other developmental stage (*p* < 0.05). Expression at L15 and at post-larval developmental stage day 1 (PL1) were 12.41-fold and 10.73-fold higher than that at L5, respectively. During the post-larval developmental stages, *Mn-GEM* gradually increased from PL5 to PL15 and peaked at PL15, which was 14.05-fold higher than that at L5 (*p* < 0.05) (Figure 3B). 

### 2.3. Expression Analysis during the Reproductive Cycle of Testes and Ovaries

**Figure 4:**
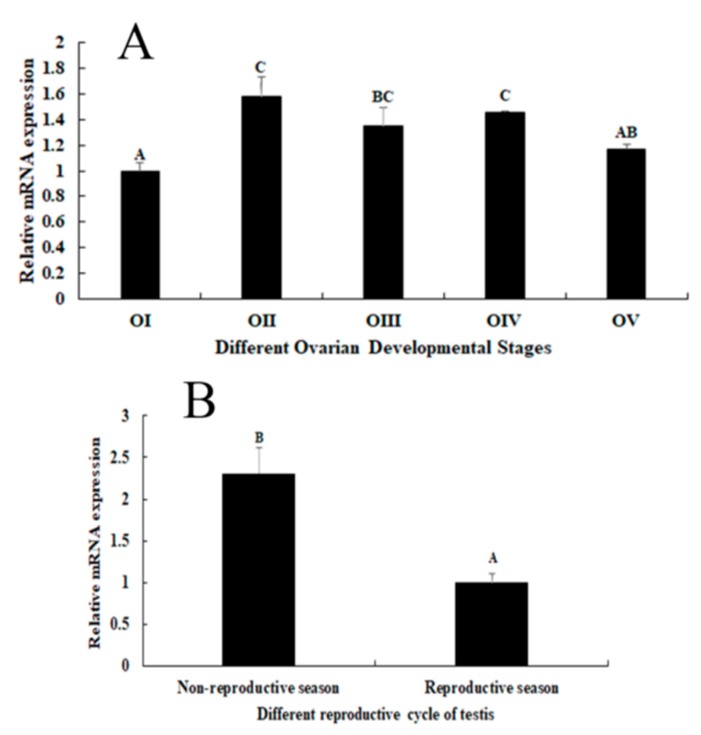
Expression characterization of *Mn-GEM* in different reproductive cycles of testis and ovary. The amount of *Mn-GEM* mRNA was normalized to the EIF transcript level. Data are shown as mean +SD (standard deviation) of tissues from three separate individuals. Capital letters indicate expression difference between different samples. (**A**) Expression characterization in different reproductive cycles of ovary. (**B**) Expression characterization in different reproductive season of testis.

Expression analysis during the various stages of the reproductive cycle of ovaries showed that *Mn-GEM* mRNA expression peaked at stage II and remained at a high level in stages III and IV. The expression levels in stages I was the lowest. Gene expression in stage II was 1.58-fold higher than that in stage I (*p* < 0.05) (Figure 4A). Gene expression in the testes during the non-reproductive season was 2.31-fold higher than that during the reproductive cycle (*p* < 0.05) (Figure 4B).

### 2.4. In situ Hybridization Analysis

**Figure 5:**
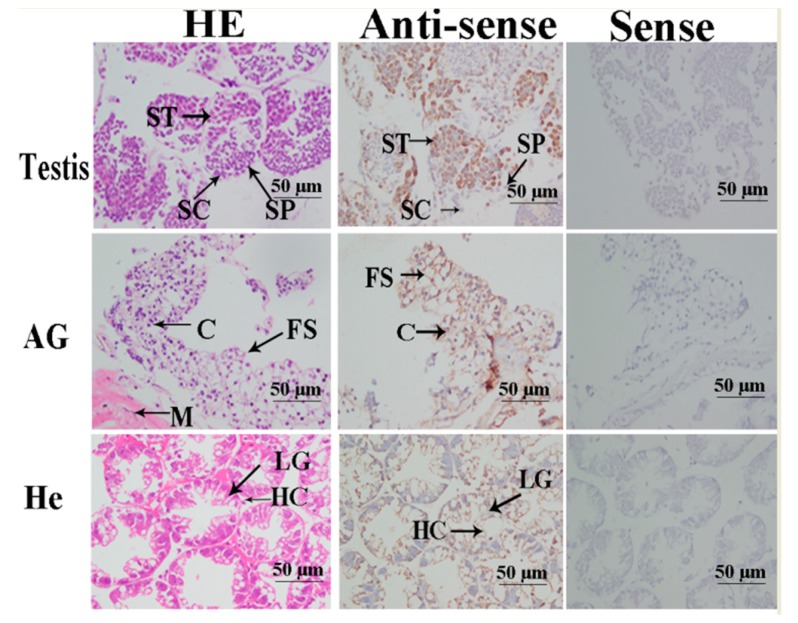
Location of the *GEM* gene was detected in testis, androgenic gland, and hepatopancreas of *M*. *nipponense* by using in situ hybridization. Testis, androgenic gland, and hepatopancreas were sampled at reproductive season. AG: androgenic gland; ST: spermatid; SC: spermatocyte; SP: sperm; M: muscle; C: androgenic gland cell; FS: funicular structure; He: hepatopancreas; LG: lipid granules; HC: hepatocytes. Scale bars = 50 μm.

**Figure 6:**
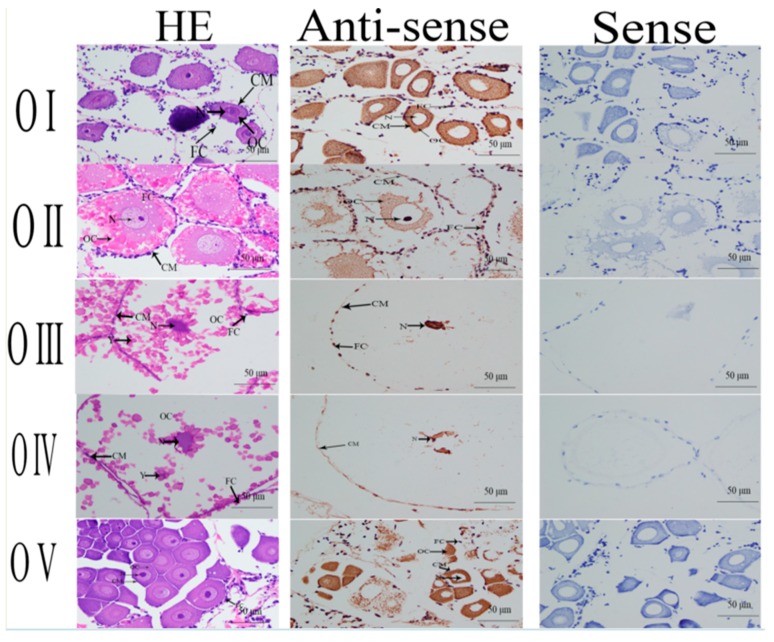
Location of the *GEM* gene was detected in ovary I to ovary V of *M*. *nipponense* by using in situ hybridization. OC: oocyte; N: nucleus; CM: cytoplasmic membrane; Y: yolk granule; FC: follicle cell. Scale bars = 50 μm.

Hematoxylin and eosin (HE) staining was used to dye the fixed tissue samples. The dominant cells in the testes during the reproductive season were sperm, although small numbers of spermatids and spermatocytes also were detected. Androgenic gland cells and funicular structure were observed in the androgenic gland, and the hepatopancreas contained lipid granules and hepatopancreas cells (Figure 5). Oogonia and follicle cells, which are derived via differentiation from ovarian epithelial cells, were observed in ovary stage I. The follicular cavity formed from the follicle cells in stage II. Oocyte volume gradually increased in stage III, and yolk granules accumulated in the oocyte (stage IV) during oogenesis and vitellogenesis (Figure 6). 

We used in situ hybridization to identify the locations of *Mn-GEM* mRNA in the androgenic gland, hepatopancreas, testes, and ovaries during different stages of the reproductive cycle. Strong signals for *GEM* were observed in spermatids in the testes only during the reproductive season. No *GEM* signal was directly observed in androgenic gland cells, but strong signals were detected in the funicular structure surrounding the androgenic gland cells. Strong *GEM* signals also were found in hepatopancreas cells, but not in lipid granules (Figure 5). Strong signals were observed in oocytes and the cytoplasmic membrane in ovary stages I, II, and V, but no signals were detected in the nucleus and follicular cells. In stages III and IV, strong signals were only observed in the nucleus (Figure 6). No signals were observed when the sense RNA probe was used. The labelling was consistent among all three independent biological replicates for each tissue.

### 2.5. RNAi Analysis

**Figure 7:**
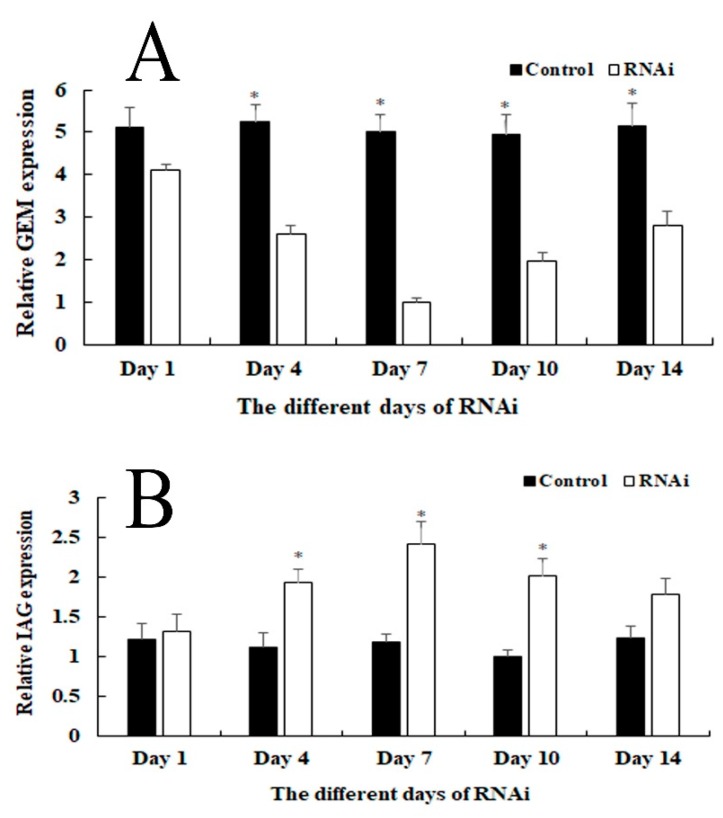
Expression characterization of *Mn-GEM* and *Mn-IAG* at different days after *Mn-GEM* dsRNA injection. The amount of *Mn-GEM* and *Mn-IAG* mRNA was normalized to the EIF transcript level. Data are shown as mean +SD (standard deviation) of tissues from three separate individuals. * indicates significant expression difference between the RNAi group and control group at the sample day. (**A**) Expression characterization of *Mn-GEM* after *Mn-GEM* dsRNA injection. (**B**) Expression characterization of *Mn-IAG* after *Mn-GEM* dsRNA injection.

RNAi analysis was performed in male prawns to evaluate the potential functions of *Mn-GEM* in male sexual differentiation and development in *M*. *nipponense* (Figure 7A). qPCR analysis revealed that *Mn-GEM* expression on different days remained stable for the control group (*p* > 0.05). However, *Mn-GEM* expression in the RNAi group gradually decreased from day 1 to 7 and reached the lowest expression level (79% relative to the control) on day 7. Expression then slightly increased until day 14. *Mn-GEM* expression levels on days 4, 7, and 10 were significantly lower in the RNAi group than in the control group (*p* < 0.05). 

We also measured Mn-insulin-like androgenic gland hormone (*Mn-IAG*) expression in the same cDNA template of the control and RNAi groups (Figure 7B). qPCR analysis revealed that *Mn-IAG* expression on different days remained stable in the control group (*p* > 0.05). However, the pattern of *Mn-IAG* expression in the RNAi group was opposite that of the *Mn-GEM* expression. *Mn-IAG* expression gradually increased from day 1 to 7 and peaked at day 7. *Mn-IAG* expression levels on days 4, 7, 10 differed significantly between the RNAi group and the control group (*p* < 0.05).

**Figure 8:**
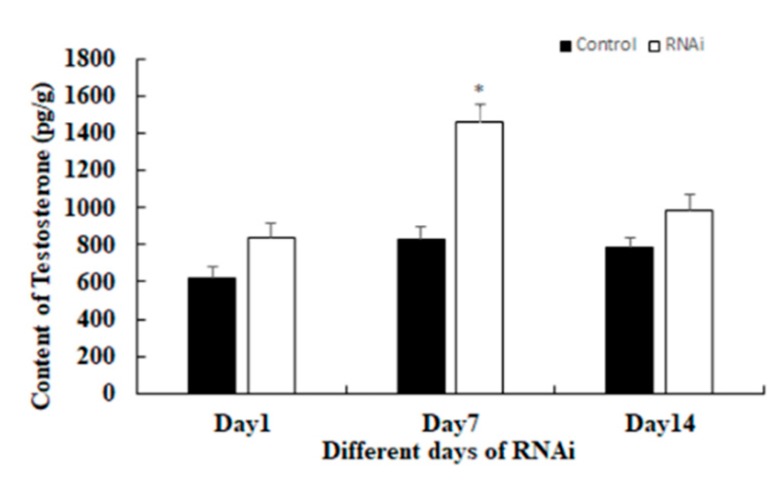
Measurement of the content of testosterone at different days after *Mn-GEM* dsRNA injection. Data are shown as mean +SD (standard deviation) of tissues from three separate individuals. * indicates significant expression difference between the RNAi group and control group at the sample day.

The content of testosterone were also measured at days 1, 7, and 14 after *Mn-GEM* dsRNA injection (Figure 8). The content of testosterone in the RNAi group was higher than that in the control group on the same day. The content of testosterone at day 7 in the RNAi group was almost two times higher than that of the control group on the same day (*p* < 0.05).

## 3. Discussion

The androgenic gland of crustaceans is important in male sexual differentiation and development, and *GEM* in this gland was predicted to be an important gene in male sexual development in *M*. *nipponense* [16]. However, the actual functions of *GEM* are still not well defined. In this study, we investigated the potential roles of *GEM* in male sexual development in *M*. *nipponense*. The full-length cDNA sequence of *Mn-GEM* was 1018 bp long and encoded 258 amino acids. Five O-GlcNAc sites were detected in the *Mn-GEM* protein. O-GlcNAc site is dynamic and controllable, which meets the demands for post-translational modification to participate in signaling pathways. O-GlcNAc site is involved in the regulation of growth, proliferation, hormone response in cells, which plays essential roles in diabetes mellitus, neurodegenerative diseases, and tumors [17]. Only one *GEM* amino acid sequence from crustacean species (*P*. *vannamei*) was submitted previously to NCBI, and *Mn-GEM* had 70% similarity with it; similarities with other species were only 37–42%. According to the phylogenetic analysis, *Mn-GEM* has the closest evolutionary relationship with *P. vannamei*. This suggests that *Mn-GEM* has little difference with that of crustacean species. However, only one *GEM* sequence of crustacean species was submitted to NCBI. The sequence difference between *GEM* of *M*. *nipponense* and other species were large, making it hard to well describe the evolutionary process of *Mn-GEM*. More *GEM* sequences from crustacean species are needed to better understand the evolutionary process of *Mn-GEM* in further studies.

To the best of our knowledge, the functions of *GEM* have not been well defined or analyzed in any species, and this is the first qPCR analysis of *GEM* expression. We performed the qPCR analysis of *GEM* in various tissues and at various developmental stages of *M*. *nipponense* as well in different stages of the reproductive cycle of the testes and ovaries. mRNA expression of *GEM* was highest in the androgenic gland, followed by the testes and ovaries, and values in these tissues were dramatically higher than those of the intestine, heart, and hepatopancreas. This finding suggests that *GEM* plays potential roles in gonad development. Additionally, *Mn-GEM* expression levels were highest at L15 and PL1 during the larval and post-larval developmental stages, indicating that it has positive effects on the metamorphosis of *M*. *nipponense* [18,19]. *Mn-GEM* expression gradually increased from PL5 to PL15 during the post-larval development, and previous studies reported that the sex differentiation sensitive period was from PL7 to PL19 [20]. The gradual increase of *Mn-GEM* expression during this time period suggested that *GEM* may play vital roles in gonad differentiation and development [13].

In situ hybridization is used to locate a specific DNA or RNA sequence in a tissue or cell using labelled complementary DNA or RNA [21]. To the best of our knowledge, no previous studies have performed in situ hybridization of *GEM* in any species. In this study, a strong *Mn-GEM* signal was detected in spermatids, which suggests that *GEM* promotes spermatid development or activates testes development in *M*. *nipponense*. In addition, *Mn-GEM* expression in the testes during the non-reproductive season was dramatically higher than that during the reproductive season. Histological observation showed that spermatids are the dominant cells in the testes during the non-reproductive season, whereas sperm dominated during the reproductive season. This result suggests that *GEM* promotes spermatid development. Strong *Mn-GEM* signals were also observed in the oocytes in ovary stages I, II, and V, indicating that it may be related to ovarian development and yolk deposition [22]. *Mn-GEM* mRNA expression peaked at ovary stage II and remained at a relatively high level in stages III and IV. Expression in stages I and V was low. These findings indicate that *Mn-GEM* plays essential roles in gametogenesis development in *M*. *nipponense*. 

Strong *Mn-GEM* signals were also detected in the funicular structure surrounding the androgenic gland cells but not in the cells themselves. A previous study reported that formation of the funicular structure indicated development of the androgenic gland, and that androgenic gland cells are subsequently formed in the funicular structure [20]. Our results indicate that *GEM* plays essential roles in the development of the funicular structure, which in turn promotes and supports formation of androgenic gland cells. The strong *Mn-GEM* signals in hepatopancreas cells suggest that it also plays important roles in the immune system of *M*. *nipponense*.

In RNAi, gene expression or translation is inhibited by short double-stranded (ds) RNA molecules in the cell’s cytoplasm [23,24,25]. RNAi has been widely used in gene function analysis in *M*. *nipponense* [14,22]. In our study, *Mn-GEM* expression in the RNAi group was significantly lower than that in the control group on the same day, indicating that dsRNA effectively inhibited *Mn-GEM* expression. To assess the regulatory roles of *GEM* in male sexual differentiation and development in *M*. *nipponense*, we also studied the regulatory relationship between *GEM* and *IAG*. *IAG*, which is a hormone secreted by and specially expressed in the androgenic gland [15], is known to play essential roles in male differentiation and development in crustacean species [26,27,28]. For example, RNAi of *IAG* resulted in significant inhibitory effects on male sexual differentiation and development of secondary sexual characteristics and spermatogenesis in *M. rosenbergii* [29]. According to our qPCR analysis, *Mn-IAG* expression increased as *Mn-GEM* expression decreased. In addition, the content of testosterone in the RNAi group was higher than that in the control group at the same day, especially on day 7. Testosterone is a steroid hormone secreted by the testis of men or the ovaries of women, as well as in a small amount from the adrenal gland. It has dramatic effects in maintaining muscle strength and quality, maintaining bone density and strength, refreshing and improving physical fitness [30,31,32]. The absence of testosterone in men can result in primary male sexual hypofunction, including Klinefelter syndrome, cryptorchidism, interstitial dysplasia or dysplasia [32,33]. Testosterone can result in sex reversal in red tilapia [34], allogynogenetic crucian carp [35,36], and the grouper *Epinephelus akaara* [37]. In crustacean species, testosterone can promote testes and sperm development [38], and Jin et al. [16] reported that testosterone is involved in early gonad differentiation and development in *M*. *nipponense*. These results suggest that *GEM* has a negative regulatory relationship with *IAG* expression and the secretion of testosterone in *M*. *nipponense*. Thus, *GEM* suppresses male sexual differentiation and development in *M*. *nipponense* by inhibiting the expression of *IAG* and the secretion of testosterone.

In conclusion, we analyzed the functions of *GEM* in *M. nipponense* using qPCR, in situ hybridization, and RNAi. Our data strongly suggest that *Mn-GEM* is involved in gonad differentiation and development, spermatid development, and gametogenesis in *M*. *nipponense*. RNAi analysis indicated that *Mn-GEM* mRNA expression has negative regulatory effects on *Mn-IAG* expression and testosterone content. These results suggest that *GEM* has reverse effects on male sexual differentiation and development in *M*. *nipponense*.

## 4. Materials and Methods

### 4.1. Sample Preparation

The samples were collected by following the previous study by our lab. Briefly, different tissues were collected from healthy adult *M*. *nipponense*, obtained from the Tai Lake in Wuxi, China (120°13’44”E, 31°28’22”N). Specimens for the different stages of larval and post-larval developmental stages were from the full-sibs population, collected with their maturation process (*N* = 5). The various phases of ovarian reproductive cycle were obtained according to the standard of previous reports (*N* = 5) [39]. The testis were collected during the reproductive season at 28 °C in summer, and during the nonreproductive season in winter at 15 °C (*N* = 5). The samples were treated with phosphate buffer saline (PBS), and immediately frozen in liquid nitrogen until use, for RNA extraction, to prevent total RNA degradation.

### 4.2. Rapid Amplification of cDNA Ends (RACE)

As described in detail previously [12,13], total androgenic gland RNA was extracted using RNAiso Plus Reagent (Takara Bio Inc., Dalian, China). The templates for 3′cDNA and 5′cDNA cloning were synthesized by using the 3′-Full RACE Core Set Ver.2.0 kit (Takara Bio Inc., Dalian, China) and the 5′-Full RACE kit (Takara Bio Inc., Dalian, China). The specific primers used for genes cloning were listed in Table 2. The BLASTX and BLASTN search program (http://www.ncbi. nlm.nih.gov/BLAST/) and the ORF Finder tool (http://www.ncbi.nlm.nih.gov/gorf/gorf.html) were employed to analyze the structural characteristics. MEGA X (https://www.megasoftware.net) was used to construct the phylogenetic trees, followed by the maximum-likelihood method with Bootstrap method of 1000 replications. 

### 4.3. qPCR Analysis

qPCR was used to measure the relative mRNA expression of *Mn-GEM* in different tissues, different developmental stages, and various reproductive cycle of testis and ovary. The Bio-Rad iCycler iQ5 Real-Time PCR System (Bio-Rad, Carlsbad, CA, USA) was used to carry out the SYBR Green RT-qPCR assay. The procedure has been well described in detail in previous studies [12,13]. The primers used for qPCR analysis are listed in Table 2. Eukaryotic translation initiation factor 5A (*EIF*) is used as the reference gene in this study [40]. The amplification efficiency between the target gene and *EIF* was determined by using different concentrations of diluted androgenic gland samples with the slope 1.393 and 1.411, respectively. 

### 4.4. In situ Hybridization of GEM

In situ hybridization was performed to analyze the mRNA locations of *Mn-GEM* in different tissues, including various reproductive cycle of ovary, the reproductive season of testis, hepatopancreas and androgenic gland (*N* = 3). Primer5 software (http://www.premierbiosoft.com/primerdesign/) was used to design the anti-sense and sense probes of CISH (chromogenic in situ hybridization) study with DIG signal based on the cDNA sequence of each gene. The sequences of anti-sense and sense probes are listed in Table 2, and are synthesized by Shanghai Sangon Biotech Company (Shanghai, China). The detailed procedures of the in situ hybridization have been well described in previous studies [13,22]. In situ hybridization experiments were performed in triplicate for each tissue, in order to measure the consistency between the independent biological replicates. Slides were examined under light microscope for evaluation.

### 4.5. RNA Interference (RNAi) Analysis

RNAi was performed to analyze the novel role of *Mn-GEM* in male sexual differentiation and development in *M*. *nipponense*. The specific RNAi primer with T7 promoter site were designed using Snap Dragon tools (http://www.flyrnai.org/cgibin/RNAifind_primers.pl) and are shown in Table 2. The Transcript Aid™ T7 High Yield Transcription kit (Fermentas, Inc, Buffalo, NY, USA) was used to synthesize the *Mn-GEM* dsRNA with the reaction conditions recommended by the manufacturer. A total of 300 healthy mature male *M*. *nipponense* with body weight of 3.4–4.6g were selected and divided into two groups, which are *GEM*-dsRNA injection (*N* = 150) and vehicle injection (*N* = 150). As described in previous study [22,41], each prawn was injected with 4 μg/g Vg dsRNA or 4 μg/g vehicles. The *GEM* mRNA expression of the androgenic gland were investigated to detect the interference efficiency by qPCR after injecting for 1, 4, 7, 10, and 14 days (*N* = 5). The *IAG* mRNA expression was investigated using the same template. 

### 4.6. Measurement of The Content of Testosterone

Testis were collected from both RNAi group and control group (*N* = 8) after injecting the ds-RNA of *Mn-GEM* for 1, 7, and 14 days. The samples were kept under −20 °C until the extraction of testosterone. Testosterone was extracted by using methyl alcohol. BECKMAN ACESS II T Kit (Brea, CA, USA) was used to measure the content of testosterone by using Beckman Coulter Access 2. All samples were run in triplicate.

### 4.7. Statistical Analysis

Quantitative data were expressed as mean ± SD. SPSS Statistics 13.0 (Chicago, IL, USA) using one-way ANOVA followed by LSD and Tukey–Kramer multiple range test measured the statistical differences for qPCR analysis in different mature tissues, developmental stages, and different reproductive cycles of ovary. Paired-samples T test was used to perform the two group comparison statistics in the same day between the RNAi group and control group. *p* < 0.05 indicates significance. 

## Figures and Tables

**Table 1:** Amino acid sequence used for phylogenetic analysis of *GEM*.

Species	Accession Number
*Macrobrachium nipponense*	
*Penaeus vannamei*	XP_027229945.1
*Acanthaster planci*	XP_022103928.1
*Xenopus laevis*	XP_018087535.1
*Stylophora pistillata*	XP_022804116.1
*Odobenus rosmarus divergens*	XP_004399834.1
*Centruroides sculpturatus*	XP_023219163.1
*Biomphalaria glabrata*	XP_013071285.1
*Aplysia californica*	XP_005105416.1
*Mizuhopecten yessoensis*	XP_021367489.1
*Xenopus tropicalis*	NP_001096228.1
*Xenopus laevis*	NP_001087945.2
*Mus musculus*	NP_079932.2
*Mus caroli*	XP_021034811.1

**Table 2:** Universal and specific primers used in this study.

Primer Name	Nucleotide Sequence (5′→3′)	Purpose
*GEM*-3GSP1	AGTATGGGAGGCTCGTCAGT	First forward primer for *GEM* 3′ RACE
*GEM*-3GSP2	ATGCCCAGTGGCTAGAAGTG	Second forward primer for *GEM* 3′ RACE
*GEM*-5GSP1	AATGCTCCAGTTCCTTTGCCT	First reverse primer for *GEM* 5′ RACE
*GEM*-5GSP2	CCTGTCGGATATGCTCCGTC	Second reverse primer for *GEM* 5′ RACE
3′ RACE OUT	TACCGTCGTTCCACTAGTGATTT	RVS first primer for 3′ RACE
3′ RACE IN	CGCGGATCCTCCACTAGTGATTTCACTATAGG	RVS second primer for 3′ RACE
5′ RACE OUT	CATGGCTACATGCTGACAGCCTA	FWD first primer for 5′ RACE
5′ RACE IN	CGCGGATCCACAGCCTACTGATGATCAGTCGATG	FWD second primer for 5′ RACE
*GEM*-RTF	ATGCCCAGTGGCTAGAAGTG	FWD primer for *GEM* expression
*GEM*-RTR	GCAGAATCCAGCGATGCTCT	RVS primer for *GEM* expression
*EIF*-F	CATGGATGTACCTGTGGTGAAAC	FWD primer for β-actin expression
*EIF*-R	CTGTCAGCAGAAGGTCCTCATTA	RVS primer for β-actin expression
*GEM* anti-sense Probe	GCACTGACGAGCCTCCCATACTACATGCTGAAGATAC	Probe for *GEM* in situ hybridization analysis
*GEM* sense Probe	GTATCTTCAGCATGTAGTATGGGAGGCTCGTCAGTGC	Probe for *GEM* in situ hybridization analysis
*GEM* RNAi-F	TAATACGACTCACTATAGGGGATATGCTCCGTCTGCACAA	FWD primer for RNAi analysis
*GEM* RNAi-R	TAATACGACTCACTATAGGGCGAATCTCATCCATCAGCAA	RVS primer for RNAi analysis
